# Monitoring antimicrobial resistance trends in commensal *Escherichia coli* from livestock, the Netherlands, 1998 to 2016

**DOI:** 10.2807/1560-7917.ES.2019.24.25.1800438

**Published:** 2019-06-20

**Authors:** Ayla Hesp, Kees Veldman, Jeanet van der Goot, Dik Mevius, Gerdien van Schaik

**Affiliations:** 1 Department of Bacteriology and Epidemiology, Wageningen Bioveterinary Research, Lelystad, the Netherlands; 2 Department of Infectious Diseases and Immunology, Faculty of Veterinary Medicine, Utrecht University, Utrecht, the Netherlands; 3 GD Animal Health, Deventer, the Netherlands; 4 Department of Farm Animal Health, Faculty of Veterinary Medicine, Utrecht University, Utrecht, the Netherlands

**Keywords:** monitoring, surveillance, antimicrobial resistance, AMR, trend analysis, quantitative, *Escherichia coli*

## Abstract

**Background:**

Monitoring of antimicrobial resistance (AMR) in animals is essential for public health surveillance. To enhance interpretation of monitoring data, evaluation and optimisation of AMR trend analysis is needed.

**Aims:**

To quantify and evaluate trends in AMR in commensal *Escherichia coli*, using data from the Dutch national AMR monitoring programme in livestock (1998–2016).

**Methods:**

Faecal samples were collected at slaughter from broilers, pigs and veal calves. Minimum inhibitory concentration values were obtained by broth microdilution for *E. coli* for 15 antimicrobials of eight antimicrobial classes. A Poisson regression model was applied to resistant isolate counts, with explanatory variables representing time before and after 2009 (reference year); for veal calves, sampling changed from 2012 represented by an extra explanatory variable.

**Results:**

Resistant counts increased significantly from 1998-2009 in broilers and pigs, except for tetracyclines and sulfamethoxazole in broilers and chloramphenicol and aminoglycosides in pigs. Since 2009, resistant counts decreased for all antimicrobials in broilers and for all but the phenicols in pigs. In veal calves, for most antimicrobials no significant decrease in resistant counts could be determined for 2009–16, except for sulfamethoxazole and nalidixic acid. Within animal species, antimicrobial-specific trends were similar.

**Conclusions:**

Using Dutch monitoring data from 1998-2016, this study quantified AMR trends in broilers and slaughter pigs and showed significant trend changes in the reference year 2009. We showed that monitoring in commensal *E. coli is* useful to quantify trends and detect trend changes in AMR. This model is applicable to similar data from other European countries.

## Introduction

Antimicrobial resistance (AMR) is recognised as one of the most urgent health issues worldwide [1-3]. Resistant bacteria emerge, evolve, persist and spread in livestock as animal reservoirs [4] selected by antimicrobial use (AMU) [5]. AMR can be transferred from animals to humans by direct contact or via the food chain and environment [4]. Therefore, monitoring of AMR in animals is an essential aspect of public health surveillance.

In 1998, a monitoring programme of AMR in livestock started in the Netherlands (NL)*.* The programme was initiated following recommendations given at the Invitational European Union (EU) Conference ‘The Microbial Threat’ hosted by the Danish Government in Copenhagen in 1998 [6]. The recommendations were ‘to monitor evolution and effects of interventions, through establishment of accurate surveillance systems on antimicrobial resistance in the human and veterinary sector’ [7]; *Escherichia coli* was chosen as the indicator organism for gut microbiota in order to monitor the effects of antimicrobials with Gram-negative spectra. Since then, results have been reported annually in the report of the Monitoring programme of antimicrobial resistance and antibiotic usage in animals in the Netherlands (MARAN) [8]. From 1998 to 2009, increasing proportions of resistant isolates were observed for several antimicrobial classes, including third generation cephalosporins and fluoroquinolones, as well as high prevalence of multidrug-resistant isolates (resistant to three or more antimicrobial classes) in broilers, slaughter pigs and veal calves [9].

These findings together with high AMU in livestock compared with other European countries resulted in drastic policy changes [9]. In 2010, the Dutch government ordered the veterinary sector to reduce overall AMU sales with 50% within 4 years. A series of mandatory targets was set, starting with a 20% AMU reduction for livestock by 2011. By 2013, an additional reduction of 30% should be observed. In 2012, this target was renewed to 70% reduction by 2015 for total livestock production. The government set 2009 as reference year for this reduction target [10]. The first two targets were achieved in 2013 through a joint effort between livestock sectors, farmers and veterinarians but the 70% target has not been fully achieved in 2018. In 2016, total antimicrobial sales for veterinary use in NL had decreased by 64% compared with 2009, as reported by the Netherlands Veterinary Medicines Institute (SDa) [11]. During this period, trends in AMR and potential effects of AMU-interventions were monitored and reported in MARAN.

So far, no formal statistical methods have been applied for trend analysis of Dutch monitoring data from livestock. Trends were typically evaluated by visual inspection of resistant proportions with confidence intervals (CIs). And to our knowledge, only a limited number of studies have been conducted to quantify trends in AMR monitoring data from livestock. For example, in 2015, a study by Hanon et al. reported resistance trends in commensal *E. coli* in the Belgium monitoring programme between 2011-14 [12]. In 2018, a descriptive trend analysis was performed by Boireau et al. to look at resistance in animal pathogens between 2002-15 [13].

Evaluation is needed of current statistical methods to optimise AMR monitoring in animals and enhance interpretation of monitoring data. The aim of this study, therefore, was to evaluate whether AMR trends could be quantified and changes detected in Dutch monitoring data from 1998 to 2016. We developed a model to quantify AMR trends over time relative to a chosen reference year in which a trend change may have occurred. Here, we describe the results of our evaluation and provide recommendations for quantitative trend analysis of AMR monitoring data.

## Methods

### Animal sampling and monitoring activities

In the Dutch monitoring programme, individual caecal samples are collected annually by the Netherlands Food and Consumer Product Safety Authority (NVWA) from broilers, pigs and veal calves in slaughterhouses. Broilers and pigs have been sampled since 1998, veal calves since 2005. Between 2005 and 2011, sampling in veal calves started with pooled faecal samples taken at farms, but from 2012 calves were sampled individually at slaughter. Since 2014, when AMR monitoring in commensal *E. coli* from livestock became mandatory by EU legislation, caecal samples have been taken from all prescribed animal species.

In the NL, ca 300 *E. coli* isolates are collected per animal species annually, which is more than the EU prescribed yearly sampling of 170 isolates per animal species. A two-stage random sampling procedure is followed to ensure that one animal per batch from one herd/flock is sampled and to minimise the risk of clustering as result of multiple samples from the same herd. First, all slaughter batches within a slaughterhouse are stratified (proportional to annual throughput of slaughtered animals) and one slaughter batch is randomly selected. Second, one animal is randomly selected from this slaughter batch for sampling.

### Bacterial isolation and susceptibility testing

The terms ‘resistant’ and ‘resistance’ in this study refer to non-wild type susceptibility, based on epidemiological cut-off (ECOFF) values as defined by The European Committee on Antimicrobial Susceptibility Testing [14]. No selective media were used to enhance detection of resistant isolates in this study. From each faecal sample, *E. coli* was isolated on MacConkey agar and one colony was randomly selected and identified as *E. coli* (biochemically by Indole test before 2012 and by matrix-assisted laser desorption/ionisation time-of-flight after 2012). Minimum inhibitory concentrations (MICs) were determined with broth microdilution, according to ISO 20776–1:2006, by commercially available microtitre plates (Sensititre EUVSEC by Thermo Scientific, East Grinstead, United Kingdom). Before antimicrobial panels were prescribed by European Food Safety Authority (EFSA) in 2008 [15] and EU-legislation in 2013, panels were periodically adjusted to improve efficiency; 10 different panels were used from 1998 to 2016. Some antimicrobials were replaced by others and MIC ranges were changed. Nevertheless, antimicrobials of relevant groups were continuously present. Amoxicillin and ampicillin were representatives of aminopenicillins; Cefotaxime and ceftazidime were representatives of cephalosporins. Gentamicin, neomycin and kanamycin were representatives of aminoglycosides. Tetracyclines were represented by doxycycline and tetracycline. Sulfamethoxazole and trimethoprim were representatives of folate pathway inhibitors. Amphenicols were represented by chloramphenicol and florfenicol. Ciprofloxacin represented the fluoroquinolones, nalidixic acid represented the quinolones.

An exception is colistin; before 2010, colistin was not in antimicrobial panels, or without sufficient MIC ranges to detect phenotypic colistin-resistance. Supplement S1, Table S1 gives an overview of panels and MIC ranges.

Between 1998 and 2016, the 12,491 isolates included in this study were collected and analysed at the Dutch National Reference Laboratory (NRL) for monitoring AMR in animals, at Wageningen Bioveterinary Research (WBVR, Lelystad, NL). Of which, 5,021 isolates were from broilers (1998–2016), 4,809 from slaughter pigs (1998–2016) and 2,651 from veal calves (2005–16). In the year 2000 no isolates were collected for any species.

### Statistical analysis of trends in resistant counts

All statistical analyses in this study were performed in R version 3.3.3 (R Foundation, Vienna, Austria). Yearly resistant isolate counts (n) were aggregated separately for each antimicrobial per species (Supplement S1, Tables S2, S3 and S4), and exact 95% confidence intervals (CIs) for the counts were calculated, using yearly total numbers of isolates tested (N). Regression models were applied using the glm() function in R and models were selected by comparison of Akaike’s Information Criterion (AIC).

The best fitting model for our purpose was a generalised linear model with Poisson distribution and a log link function (Poisson regression) for yearly resistance counts (n), with the log of the total number of strains per year (N) as offset. In our model, trends in AMR were modelled relative to a reference year for all animal species, to specifically test whether a trend change was observed. Two explanatory numerical variables were used: ‘time in years 1998-2009 until start of AMU interventions’ (x1) and ‘time in years 2009-2016 since start of AMU interventions’ (x2). The notation of the x-variables were:

x1 Time in years until reference year: -11,-10,-9,-8,-7,-6,-5,-4,-3,-2,-1, 0, 0, 0, 0, 0, 0, 0, 0

x2 Time in years since reference year: 0, 0, 0, 0, 0, 0, 0, 0, 0, 0, 0, 0, 1, 2, 3, 4, 5, 6, 7

The chosen reference year in the model was ‘0’ in both explanatory variables, making this year the model intercept and the estimate for the mean resistant proportion in the year 2009. Estimates for x1 and x2 indicated whether a significant trend change occurred. The exponent of the estimates gave incidence rate ratios (IRRs), which quantified the mean increase or decrease per year, an IRR of 1 indicating the mean change of the resistant proportion per year is zero (no trend), an IRR > 1 indicating a mean increase over time and an IRR < 1 a mean decrease over time. This specific notation made the model flexible to analyse trend changes. By varying x1 and x2 and comparing model fit, we could also assess in which year a trend change had most likely taken place. Only results with 2009 as reference year are presented here, because this was set as index year by the government and to measure AMU reduction by the Netherlands Veterinary Medicines Institute [11] and was sufficient to illustrate our method. The 95% CIs for IRRs were calculated as were CIs for predicted values, using the inverse link-function.

To verify our method, we compared it with a generalised linear model with binomial distribution, using the same notation for x1 and x2. The Poisson model had lower AICs for most data. Goodness-of-fit was tested using the deviance chi-squared goodness-of-fit test, and assessing scaled deviances (with a dispersion parameter of 1; a scaled deviance of > 2 indicated overdispersion and a scaled deviance of < 0.5 indicated underdispersion). With overdispersion, the variance of the count is much larger than the mean, a common problem with count data. For a few antimicrobial-species combinations model fit was suboptimal i.e. Poisson’s assumptions were not met. For cases with over/underdispersed data, a negative binomial or binomial distribution was applied, respectively, to improve model fit.

As the sampling of veal calves changed in 2011, an extra variable was added to the model to detect possible effects of this sampling change. This variable x3 was ‘0’ until 2011 and ‘1’ from 2012.

Colistin resistance data was only available since 2010 (Supplement S1, Table S1), and was analysed with Poisson regression for 2010–16 for broilers and slaughter pigs, with ‘x’ representing time in years.

The following antimicrobials from the same class for which *E. coli* is considered to be cross-resistant: amoxicillin/ampicillin and doxycycline/tetracycline and neomycin/kanamycin [16] were modelled as if being equal.

## Results

### Broilers

**Figure 1:**
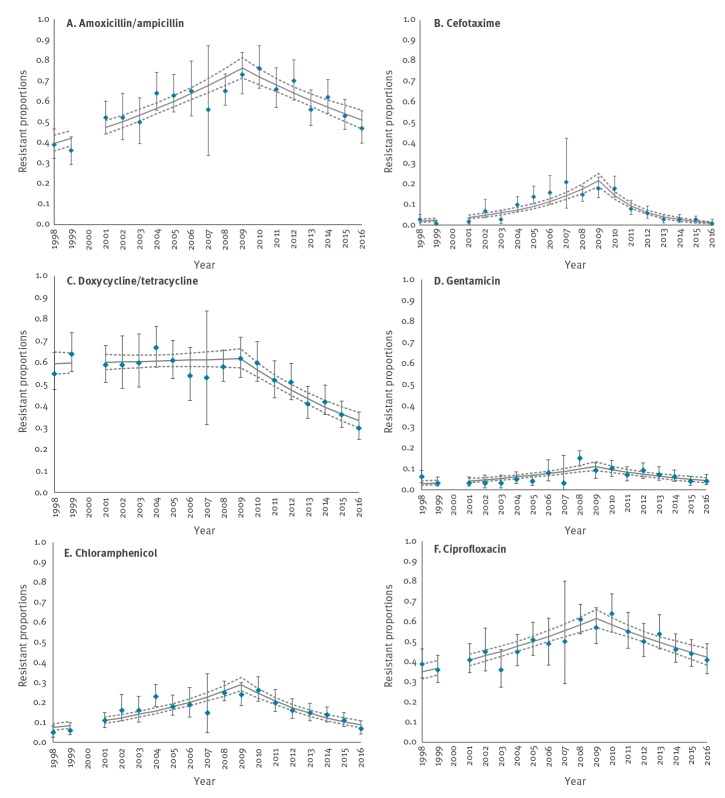
Resistant proportions in isolates from broilers, modelled as resistant counts with Poisson regression and time in years before^a^ and after^b^ 2009, the Netherlands, 1998–2016

^a^ Time in years 1998–2009 (x1).

^b^ Time in years 2009–16 (x2).

Between 1998 and 2009 (x1), there were statistically significant increasing resistance trends for all antimicrobials (range IRR: 1.04–1.30), except for tetracyclines (IRR: 1.0; 95% CI: 0.99–1.02; p = 0.56) and sulfamethoxazole (IRR: 1.0; 95% CI: 0.98–1.03; p = 0.69). Between 2009 and 2016 (x2), significant decreasing resistance trends were observed for all antimicrobials in broilers (range IRR: 0.66–0.95) (Table 1, Figure 1).

**Table 1:** Estimates for antimicrobial resistance trends in yearly resistant counts of *Escherichia coli* from broilers, for time in years before (1998–2009)^a^ and after (2009–2016)^b^ antimicrobial use interventions, the Netherlands, 1998–2016

Antimicrobial	Variable	Estimate	P value^c^	IRR^d^ (95% CI)	Scaled deviance^e^ (0.5 < > 2)
Amoxicillin/ampicillin	Intercept	- 0.27	0.00	0.76 (0.71–0.81)	1.21
	x1	0.06^a^	0.00	1.06 (1.05–1.07)	
	x2	- 0.06^b^	0.00	0.94 (0.93– 0.96)	
Cefotaxime	Intercept	- 1.53	0.00	0.22 (0.19–0.25)	2.68
	x1	0.21	0.00	1.24 (1.19–1.29)	
	x2	- 0.42	0.00	0.66 (0.61–0.71)	
Ceftazidime^f^	Intercept	- 1.59	0.00	0.20 (0.17–0.24)	1.55
	x1	0.24	0.00	1.27 (1.20–1.35)	
	x2	- 0.39	0.00	0.67 (0.63–0.72)	
Gentamicin	Intercept	- 2.20	0.00	0.11 (0.09–0.13)	1.93
	x1	0.12	0.00	1.13 (1.08–1.17)	
	x2	- 0.13	0.00	0.88 (0.83–0.92)	
Doxycycline/tetracycline	Intercept	- 0.48	0.00	0.62 (0.57–0.66)	0.63
	x1	0.00	0.56	1.00 (0.99–1.02)	
	x2	- 0.09	0.00	0.91 (0.90–0.93)	
Sulfamethoxazole^f^	Intercept	- 0.31	0.00	0.73 (0.68–0.79)	0.39
	x1	0.01	0.69	1.01 (0.98–1.03)	
	x2	- 0.08	0.00	0.93 (0.91–0.95)	
Trimethoprim	Intercept	- 0.42	0.00	0.66 (0.61–0.70)	1.41
	x1	0.04	0.00	1.04 (1.02–1.05)	
	x2	- 0.08	0.00	0.92 (0.90–0.94)	
Chloramphenicol	Intercept	- 1.24	0.00	0.29 (0.26–0.32)	1.52
	x1	0.12	0.00	1.13 (1.10–1.16)	
	x2	- 0.17	0.00	0.84 (0.81–0.87)	
Florfenicol^f^	Intercept	- 3.02	0.00	0.05 (0.03–0.07)	1.49
	x1	0.27	0.00	1.30 (1.19–1.45)	
	x2	- 0.31	0.00	0.73 (0.60–0.89)	
Ciprofloxacin	Intercept	- 0.49	0.00	0.61(0.57–0.66)	0.69
	x1	0.05	0.00	1.05 (1.04–1.07)	
	x2	- 0.05	0.00	0.95 (0.93–0.97)	
Nalidixic acid^f^	Intercept	- 0.46	0.00	0.63 (0.58–0.69)	0.59
	x1	0.06	0.00	1.06 (1.03–1.10)	
	x2	- 0.07	0.00	0.94 (0.92–0.96)	
Neomycin/kanamycin^f^	Intercept	- 1.85	0.00	0.16 (0.13–0.19)	1.25
	x1	0.05	0.01	1.05 (1.01–1.10)	
	x2	- 0.21	0.00	0.81 (0.73–0.89)	

*E. coli: Escherichia coli;* CI: confidence interval; IRR: incidence rate ratio.

^a^ Estimate for antimicrobial resistance trends in years 1998–2009.

^b^ Estimate for antimicrobial resistance trends in years 2009–­16.

^c^ P values < 0.05 indicate significant trends for variables x1 (1998-2009) and x2 (2009-16).

^d^ IRR with 95% CI: for the intercept this number indicates the estimated resistant proportion for reference year 2009. For variables x1 and x2 this is the mean increase or decrease per year.

^e^ Scaled deviance of > 2 indicates overdispersion of data, scaled deviance of < 0.5 indicates underdispersion of data.

^f^ Data were not collected during whole length of testing period, see Supplement S1, Table S1.

Vertical error bars indicate 95% confidence intervals (CIs) per yearly observation. Model predicted values are visualised in the grey line with their 95% CIs (grey dotted lines).

Additional analyses with the same modelling approach but using 2010 as reference year instead of 2009 showed that for most antimicrobials the decreasing trend started after 2010; the model with 2010 as reference year had a better fit (data not shown). However, overall, the model fit with 2009 as reference year was good reflected by the scaled deviances in Table 1 and CIs in Figure 1. The cefotaxime data was overdispersed (scaled deviance 2.68); a negative binomial distribution was applied, which better fit the data (scaled deviance 1.32) and gave similar estimates.

### Slaughter pigs

The observed resistant counts were generally lower than in broilers, except for tetracyclines; the estimated resistant proportion for 2009 was 0.72 (0.67–0.77) (Table 2).

**Table 2:** Estimates for antimicrobial resistance trends in yearly resistant counts of *Escherichia coli* from slaughter pigs, for time in years before (1998–2009)^a^ and after (2009–2016)^b^ antimicrobial use interventions, the Netherlands, 1998–2016

Antimicrobial	Variable	Estimate	P value^c^	IRR^d^ (95% CI)	Scaled deviance^e^ (0.5 < > 2)
Amoxicillin/ampicillin	Intercept	- 0.91	0.00	0.40 (0.37–0.44)	1.17
	x1	0.09^a^	0.00	1.09 (1.07–1.11)	
	x2	- 0.09^b^	0.00	0.91 (0.89–0.94)	
Cefotaxime	Intercept	-3.98	0.00	0.02 (0.01–0.03)	1.39
	x1	0.15	0.01	1.16 (1.05–1.30)	
	x2	- 0.23	0.01	0.79 (0.66–0.93)	
Ceftazidime^f^	Intercept	- 3.83	0.00	0.02 (0.01–0.03)	1.92
	x1	0.17	0.01	1.18 (1.05–1.39)	
	x2	- 0.23	0.00	0.80 (0.67–0.93)	
Gentamicin	Intercept	- 3.49	0.00	0.03 (0.02–0.04)	2.57
	x1	0.13	0.00	1.14 (1.06–1.24)	
	x2	- 0.16	0.01	0.85 (0.76–0.95)	
Doxycycline/tetracycline	Intercept	- 0.33	0.00	0.72 (0.67–0.77)	0.72
	x1	0.03	0.00	1.04 (1.02–1.05)	
	x2	- 0.08	0.00	0.93 (0.91–0.95)	
Sulfamethoxazole^f^	Intercept	- 0.51	0.00	0.60 (0.55–0.66)	0.20
	x1	0.03	0.04	1.03 (1.00–1.07)	
	x2	- 0.08	0.00	0.93 (0.91–0.95)	
Trimethoprim	Intercept	- 0.65	0.00	0.52 (0.48–0.57)	0.71
	x1	0.04	0.00	1.04 (1.02–1.05)	
	x2	- 0.08	0.00	0.92 (0.90–0.95)	
Chloramphenicol	Intercept	-2.18	0.00	0.11 (0.10–0.13)	1.12
	x1	0.03	0.07	1.03 (1.00–1.06)	
	x2	0.00	0.97	1.00 (0.96–1.05)	
Florfenicol^f^	Intercept	- 4.61	0.00	0.01 (0.004–0.02)	1.15
	x1	0.22	0.02	1.25 (1.05–1.54)	
	x2	0.09	0.56	1.09 (0.81–1.46)	
Ciprofloxacin	Intercept	- 3.39	0.00	0.03 (0.02–0.05)	3.48
	x1	0.15	0.00	1.16 (1.07–1.27)	
	x2	- 0.43	0.00	0.65 (0.54–0.77)	
Nalidixic acid^f^	Intercept	- 3.25	0.00	0.04 (0.02–0.06)	3.29
	x1	0.36	0.00	1.43 (1.16–1.82)	
	x2	- 0.46	0.00	0.63 (0.52–0.76)	
Neomycin/kanamycin^f^	Intercept	- 3.33	0.00	0.04 (0.02–0.05)	1.08
	x1	0.04	0.37	1.04 (0.95–1.14)	
	x2	- 0.47	0.00	0.62 (0.46–0.81)	

*E. coli: Escherichia coli;* CI: confidence interval; IRR: incidence rate ratio.

^a^ Estimate for antimicrobial resistance trends in years 1998–2009.

^b^ Estimate for antimicrobial resistance trends in years 2009–­16.

^c^ P values < 0.05 indicate significant trends for variables x1 (1998-2009) and x2 (2009-16).

^d^ IRR with 95% CI: for the intercept this number indicates the estimated resistant proportion for reference year 2009. For variables x1 and x2 this is the mean increase or decrease per year.

^e^ Scaled deviance of > 2 indicates overdispersion of data, scaled deviance of < 0.5 indicates underdispersion of data.

^f^ Data were not collected during whole length of testing period, see Supplement S1, Table S1.

Between 1998 and 2009 (x1), there were statistically significant increasing resistance trends for all antimicrobials (range IRR: 1.03–1.43), except for chloramphenicol (IRR: 1.03; 95% CI: 1.00–1.06; p = 0.07). Between 2009 and 2016 (x2), significant decreasing resistance trends were observed for all antimicrobials in pigs (range IRR: 0.66–0.95), with exception of chloramphenicol- (IRR: 1.00; 95% CI: 0.96-1.05; p = 0.97) and florfenicol resistance (IRR: 1.09; 95% CI: 0.81–1.46; p = 0.56) (Table 2).

For quinolones and gentamicin, model fit was suboptimal, data were overdispersed. A model with a negative binomial distribution resulted in different estimates for quinolones and similar estimates for gentamicin (Supplement S1, Tables S5).

### Veal calves

Results showed that trends between 2005 and 2009 (x1), and between 2009 and 2016 (x2), could not be analysed without taking into account variable x3, a binary variable representing the sampling change from 2012 (Table 3). When variable x3 was added, the fit increased significantly for all antimicrobials (Table 3). AICs improved and overdispersion was reduced for gentamicin, trimethoprim and quinolones (Table 3). Collinearity between x2 and x3 was not considered a problem; standard errors of explanatory variables were not greatly influenced by adding x3, and Variance Inflation Factors of x-variables were acceptable.

**Table 3:** Estimates for antimicrobial resistance trends in yearly resistant counts of *Escherichia coli* from veal calves, for time in years before^a^ and after^b^ antimicrobial use interventions, with and without including a sampling change as extra explanatory variable^c^, the Netherlands, 2005–2016

Antimicrobial	Variable	Estimate	P value^d^	IRR^e^ (95% CI)	AIC^f^	Scaled deviance^g^ (0.5 < > 2)
Amoxicillin/ampicillin	Intercept	- 0.83	0.00	0.44 (0.38–0.50)	97.09	1.97
	x1	- 0.03^a^	0.41	0.97 (0.92–1.04)		
	x2	- 0.12^b^	0.00	0.89 (0.86–0.92)		
	Intercept	- 0.82	0.00	0.44 (0.38–0.50)	87.11	0.72
	x1	- 0.02^a^	0.44	0.98 (0.92–1.04)		
	x2	- 0.02^b^	0.62	0.98 (0.92–1.05)		
	x3	- 0.56^c^	0.00	0.57 (0.41–0.78)		
Cefotaxime	Intercept	- 3.65	0.00	0.03 (0.01–0.05)	47.49	0.99
	x1	- 0.03	0.79	0.97 (0.75–1.26)		
	x2	- 0.32	0.00	0.72 (0.58–0.88		
	Intercept	- 3.71	0.00	0.02 (0.01–0.05)	47.60	0.87
	x1	- 0.06	0.67	0.95 (0.86–1.64)		
	x2	- 0.07	0.73	0.93 (0.61–1.39)		
	x3	- 1.30	0.18	0.27 (0.04–1.72)		
Ceftazidime	Intercept	- 3.65	0.00	0.03 (0.01–0.05)	45.49	1.54
	x1	0.15	0.34	1.17 (0.86–1.64)		
	x2	- 0.44	0.00	0.64 (0.49–0.81)		
	Intercept	- 3.88	0.00	0.02 (0.01–0.04)	41.71	1.01
	x1	0.08	0.65	1.08 (0.79–1.52)		
	x2	0.10	0.69	1.11 (0.67–1.83)		
	x3	- 2.85	0.02	0.06 (0.00–0.60)		
Gentamicin	Intercept	- 2.37	0.00	0.09 (0.07–0.12)	88.38	3.51
	x1	- 0.05	0.44	0.95 (0.83–1.08)		
	x2	- 0.24	0.00	0.79 (0.72–0.86)		
	Intercept	- 2.46	0.00	0.09 (0.06–0.11)	73.54	1.85
	x1	- 0.08	0.23	0.92 (0.81–1.05)		
	x2	0.11	0.26	1.11 (0.92–1.34)		
	x3	- 1.83	0.00	0.16 (0.06–0.39)		
Tetracycline	Intercept	- 0.39	0.00	0.68 (0.61–0.75)	100.22	1.60
	x1	- 0.04	0.12	0.96 (0.92–1.01)		
	x2	- 0.08	0.00	0.93 (0.90–0.95)		
	Intercept	- 0.38	0.00	0.68 (0.61–0.75)	94.98	0.89
	x1	- 0.03	0.15	0.97 (0.92–1.01)		
	x2	- 0.02	0.42	0.98 (0.93–1.03)		
	x3	- 0.33	0.01	0.72 (0.57–0.91)		
Sulfamethoxazole	Intercept	- 0.65	0.00	0.52 (0.46–0.59)	96.57	1.71
	x1	- 0.01	0.83	0.99 (0.94–1.05)		
	x2	- 0.12	0.00	0.88 (0.86–0.91)		
	Intercept	- 0.65	0.00	0.52 (0.46–0.59)	93.56	1.30
	x1	0.00	0.87	1.00 (0.94–1.05)		
	x2	- 0.06	0.04	0.94 (0.88–1.00)		
	x3	- 0.33	0.03	0.72 (0.53–0.96)		
Trimethoprim	Intercept	- 0.82	0.00	0.44 (0.38–0.50)	98.43	2.23
	x1	0.00	0.98	1.00 (0.94–1.07)		
	x2	- 0.14	0.00	0.87 (0.84–0.90)		
	Intercept	- 0.82	0.00	0.44 (0.38–0.50)	92.63	1.53
	x1	0.00	0.96	1.00 (0.94–1.07)		
	x2	- 0.06	0.11	0.94 (0.88–1.01)		
	x3	- 0.47	0.01	0.63 (0.45–0.87)		
Chloramphenicol	Intercept	- 1.44	0.00	0.24 (0.20–0.28)	90.66	1.96
	x1	- 0.07	0.07	0.93 (0.86–1.01)		
	x2	- 0.10	0.00	0.91 (0.87–0.95)		
	Intercept	- 1.43	0.00	0.24 (0.20–0.28)	82.09	0.88
	x1	- 0.07	0.08	0.93 (0.87–1.01)		
	x2	0.03	0.56	1.03 (0.94–1.12)		
	x3	- 0.70	0.00	0.50 (0.32–0.76)		
Florfenicol^h^	Intercept	- 1.99	0.00	0.14 (0.10–0.18)	83.13	5.53
	x1	0.00	0.96	1.00 (0.90–1.13)		
	x2	- 0.14	0.02	0.87 (0.77–0.97)		
	Intercept	- 2.22	0.00	0.11 (0.08–0.15)	67.79	3.17
	x1	- 0.07	0.25	0.93 (0.82–1.05)		
	x2	0.29	0.01	1.33 (1.06–1.68)		
	x3	- 1.53	0.00	0.22 (0.11–0.45)		
Ciprofloxacin	Intercept	- 1.55	0.00	0.21 (0.17–0.26)	90.42	2.66
	x1	0.00	0.96	1.00 (0.91–1.09)		
	x2	- 0.25	0.00	0.78 (0.73–0.82)		
	Intercept	- 1.58	0.00	0.21 (0.17–0.25)	80.78	1.54
	x1	- 0.01	0.77	0.99 (0.90–1.08)		
	x2	- 0.06	0.34	0.94 (0.83–1.07)		
	x3	-1.00	0.00	0.37 (0.20–0.66)		
Nalidixic acid	Intercept	-1.54	0.00	0.21 (0.17–0.26)	90.80	2.83
	x1	0.00	0.98	1.00 (0.91–1.10)		
	x2	- 0.29	0.00	0.75 (0.70–0.80)		
	Intercept	- 1.57	0.00	0.21 (0.17–0.26)	86.34	2.37
	x1	- 0.01	0.82	0.99 (0.90–1.09)		
	x2	- 0.14	0.04	0.87 (0.76–1.00)		
	x3	- 0.78	0.01	0.46 (0.25–0.84)		
Neomycin/kanamycin^h^	Intercept	- 1.65	0.00	0.19 (0.15–0.24)	105.25	8.63
	x1	- 0.04	0.42	0.96 (0.87–1.06)		
	x2	- 0.20	0.00	0.82 (0.74–0.90)		
	Intercept	- 1.91	0.00	0.15 (0.11–0.19)	74.76	3.86
	x1	- 0.13	0.02	0.88 (0.79–0.98)		
	x2	0.30	0.00	1.35 (1.11–1.64)		
	x3	- 1.86	0.00	0.16 (0.08–0.29)		

AIC: Akaike’s Information Criterion; *E. coli: Escherichia coli;* CI: confidence interval; IRR: incidence rate ratio.

^a^ Estimate for antimicrobial resistance trends in years 2005–09.

^b^ Estimate for antimicrobial resistance trends in years 2009–­16.

^c^ 0/1 variable for the change in sampling from 2012 onwards, from then individual animal samples were taken at slaughterhouses, from 2005-2011 floor samples were taken on veal farms.

^d^ P values < 0.05 indicate significant trends for variables x1 (2005-09) and x2 (2009-16).

^e^ IRR with 95% CIs, for the intercept this number indicates the estimated resistant proportion for reference year 2009. For variables x1 and x2 this is the mean increase or decrease per year.

^f^ A measure for the fit of the model, lower values for the model including x3 indicate a better fit.

^g^ Scaled deviance of > 2 indicates overdispersion of data, scaled deviance of < 0.5 indicates underdispersion of data.

^h^ Data were not collected during whole length of testing period, see Supplement S1, Table S1.

**Figure 2:**
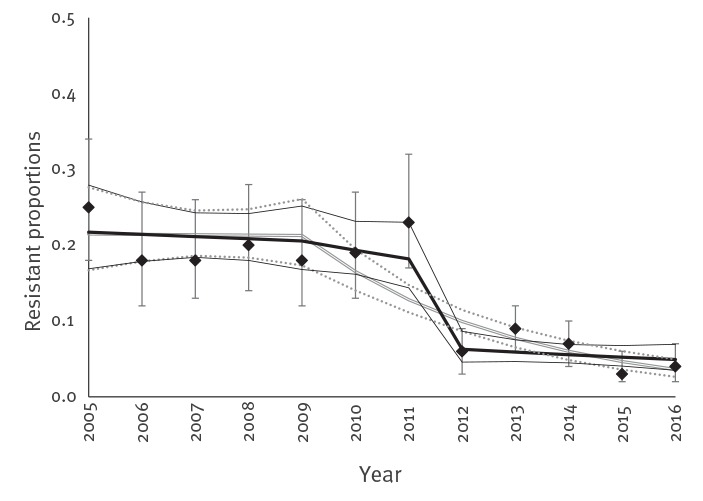
Resistant proportions per year for ciprofloxacin in isolates from veal calves, modelled as resistant isolate counts with Poisson regression and time in years before^a^ and after 2009^b^, with and without including the variable for the sampling change from 2012 onwards^c^, the Netherlands, 2005–2016

^a^ Time in years 1998–2009 (x1).

^b^ Time in years 2009–16 (x2).

^c^ 0/1 variable for the change in sampling from 2012 onwards (x3).

When taking into account the sampling change from 2012, no significant decreasing trend could be estimated in the monitoring data from 2009 to 2016 in veal calves for all antimicrobials except sulfamethoxazole and naladixic acid (Table 3). In 2012, a sharp decrease in resistant counts was observed, due to the change in sampling strategy, explained by x3, illustrated for ciprofloxacin in Figure 2. For nalidixic acid, florfenicol and aminoglycosides, data were overdispersed (Table 3). A model with negative binomial distribution better fit these data, resulting in similar estimates as the Poisson model (data not shown). Modelling a subset of the veal calves data from 2012 to 2016, only resulted in significant decreases for sulfamethoxazole, trimethoprim, ciprofloxacin and nalidixic acid with IRRs of 0.90, 0.91, 0.85 and 0.76, respectively (data not shown).

Vertical error bars indicate 95% confidence intervals (CIs) per yearly observation. The black bold line presents predicted values for the model including the variable for sampling change (x3), with the model’s 95% CIs (black thin lines). The grey line in the background presents predicted values for the model without the variable for sampling change (x3), with the model’s 95% CIs (grey dotted lines).

### Colistin resistance

Colistin-resistant isolates were detected sporadically in Dutch monitoring programme data since 2010. Time trends for colistin can be seen in Table 4. No significant decrease was detected for slaughter pigs, however for broilers a significant decreasing trend was observed. For veal calves, a decreasing trend could not be distinguished reliably from the effect of the sampling change (data not shown).

**Table 4:** Results of Poisson regression of antimicrobial resistance trends in yearly resistant counts of indicator *E. coli* resistant to colistin from broilers and slaughter pigs, the Netherlands, 2010–2016

Species	Year	n	Resistant Count (n)		Estimate	P value^a^	IRR^b^ (95% CI)	AIC^c^	Scaled deviance^d^ (0.5 < > 2)
Broilers	2010	284	3	Intercept	- 3.80	0.00	0.02 (0.01–0.06)	22.57	1.40
	2011	283	2	x	- 0.46	0.01	0.63 (0.42–0.88)		
	2012	292	3						
	2013	301	3						
	2014	377	0						
	2015	400	0						
	2016	300	0						
Slaughter pigs	2010	282	0	Intercept	- 4.92	0.00	0.01 (0.00–0.05)	14.31	1.14
	2011	287	2	x	- 0.53	0.16	0.59 (0.23–1.12)		
	2012	284	1						
	2013	289	0						
	2014	392	0						
	2015	298	0						
	2016	299	0						

AIC: Akaike’s Information Criterion; *E. coli: Escherichia coli;* CI: confidence interval; IRR: incidence rate ratio.

^a^ P values < 0.05 indicate significant trends for variables x.

^b^ IRR with 95% CIs, for the intercept this number indicates the estimated resistant proportion for 2010. For variable x this is the mean decrease per year.

^c^ A measure for the fit of the model, a lower value indicates a better fit.

^d^ Scaled deviance of > 2 indicates overdispersion of data, scaled deviance of < 0.5 indicates underdispersion of data.

## Discussion

This study aimed to optimise interpretation of AMR monitoring data by modelling resistance trends in commensal *E. coli* from livestock and to evaluate if any trends (and trend changes) were observed from 1998 to 2016. We developed a model that optimised the quantification of resistance trends and the detection of trend changes as a likely effect of interventions in indicator commensal *E. coli* from livestock. We conclude that monitoring in indicator commensal *E.coli* is valuable to evaluate resistance trends in livestock on animal population level. For nearly all antimicrobials in broilers and slaughter pigs, significant and quantifiable changes were observed in NL monitoring from 1998 to 2016. Significant decreases since 2009 were mostly preceded by significant increases from 1998 to 2009 and there was high similarity in trends for all antimicrobials within animal species.

### Broilers

An increasing veterinary therapeutic AMU was measured in NL between 1998 and 2009 [11], corresponding to the AMR trends we found in the broiler data over this time period. For most antimicrobials resistant proportions started to decrease from 2010, confirmed in an additional analysis by the better fit of broiler data in models with 2010 as reference year (data not shown). In 2010, the illegal prophylactic use of ceftiofur on day-old chicks in hatcheries ended following intensified control measures implemented by the Dutch Food Safety Authority. This may have resulted in the abrupt and significant decreases of cefotaxime- and ceftazidime resistant counts after 2010. Interestingly, however, the observed resistant proportions for ciprofloxacin in broilers remained high and although these proportions decreased significantly since 2009, it is at a slower rate than expected.

Fluoroquinolone-use has decreased considerably in broilers since 2009 [17]. As part of the intervention measures, fluoroquinolone-use in livestock was legally restricted as was the use of third generation cephalosporins. Since January 2014, these antimicrobials are only allowed to be used after veterinarians have confirmed by antibiogram that no alternative antibiotics are available (with exception of ceftiofur, which was never licensed in poultry) [9]. The relative persistence of ciprofloxacin-resistant *E. coli* in broilers may be explained by chromosomal mutations, which have a low bacterial fitness cost [18]; ciprofloxacin-resistance is mostly not encoded on plasmids like cefotaxime-resistance is. It is speculated that ciprofloxacin-resistance may be transmitted between broiler flocks, or be introduced from parent stocks, from the farm environment or from hatcheries but it is currently unclear so further investigations are needed. Persistence of quinolone-resistance in livestock is very relevant since fluoroquinolones are marked as critically important antimicrobials by WHO [19].

### Slaughter pigs

From 1998 to 2009, resistant counts increased in slaughter pigs, except for chloramphenicol and for the aminoglycosides. Resistant proportions of *E. coli* isolates decreased significantly since 2009 for all antimicrobials, except chloramphenicol and florfenicol; corresponding to data from the Netherlands Veterinary Medicines Institute who also observed an AMU decrease since 2009 [11]. In general, resistant proportions were lower in isolates from slaughter pigs than in broilers, with exception of tetracycline-resistance. Despite the fact that chloramphenicol has not been used in pigs since its ban in the early 1990s, resistance remained and has not decreased since 2009; the frequent use of florfenicol in pigs may be the cause of this as florfenicol selects for the presence of *floR* genes, which confers resistance to both chloramphenicol and florfenicol [16]. Furthermore, co-selection of *cat*-genes in Class 1 integrons by other substances (tetracyclines, aminoglycosides, sulfonamides or trimethoprim) as described by Wu et al. may explain this phenomenon [20]. Tetracycline use in pigs has decreased since 2009, but is still relatively high [11].

For gentamicin and quinolones, overdispersion of data in the Poisson model hampered trend analysis. For antimicrobials of which resistant proportions are nearly zero and when the data has many zero counts, determining aberrations in trends can be difficult. In general, with a negative binomial distribution these trends could still be assessed reliably in this study.

### Veal calves

Changing from pooled samples from farms to individual animals at slaughter had a large impact on observed resistant counts in veal calves. Trends could not be assessed without including this sampling change in the model. We conclude that in spite of a substantial decrease in total AMU in veal calves from 2007 to 2015 (as reported by the SDa [11]) for most antimicrobials no significant decrease in resistant proportions of *E. coli* could be determined with the current monitoring system from 2009 to 2016, except for sulfamethoxazole and nalidixic acid. Looking specifically at the trend from 2012 to 2016, after the sampling change, a significant decreasing trend was observed for quinolones, sulfamethoxazole and trimethoprim, but not for other antimicrobials. Between 2005 and 2009, for most antimicrobials in veal calves no significant trend was observed.

### Colistin as example of trend analysis in rare resistance

Quantifying trends in resistant isolates from livestock is needed to support treatment guidelines and AMR policy. When resistance is non-existent or rare, monitoring with a limited number of samples may not be able to detect emerging resistance, or resistance with a low prevalence. The statistical model used in this study was appropriate with a yearly sample of 300 isolates. Although we did not test it explicitly, this result for colistin may indicate that yearly sampling of 170 isolates, as currently prescribed by EFSA [15], may not be sensitive enough to detect changes in rare resistance traits especially when changes are small. The effect of different sampling strategies in the monitoring on both detecting emerging resistance and trend changes should be further investigated.

### Commensal *Escherichia coli* as sentinel organism

Often, resistant proportions in sentinel organism *E. coli* are referred to as ‘prevalence’ of resistance. However, *E. coli* is only a minor fraction of gut microbiota and detected resistant proportions cannot be translated directly to AMR prevalence in livestock in general [21]. Nonetheless, commensal *E. coli* can be used as an indicator organism to study AMR-trends in Gram-negative bacteria in livestock, which are intrinsically susceptible to the antimicrobials used in the panel. Because *E. coli* is present in all faecal samples, randomisation of sampling is possible. Furthermore, the wildtype is susceptible to all of the tested antimicrobials and isolation methods for *E. coli* from animal faeces can be standardised. These are the characteristics which make *E. coli* a useful indicator. This study stresses that when standardised AMR monitoring in *E. coli* is performed continuously, time-trends can be analysed reliably. These trends indicate if AMU interventions are necessary and when measures are taken their effect on monitored resistant counts is reflected in the monitoring data.

### Changes of antimicrobial panel

In the analysis, resistant proportions for amoxicillin/ampicillin, doxycycline/tetracycline and neomycin/kanamycin were modelled as if being one. This enabled trend analysis for these antimicrobials and significant decreasing trends were shown in both broilers and slaughter pigs. For amoxicillin and ampicillin, resistance in *E. coli* is encoded by the same genes and the same resistance mechanisms are involved [16]. Doxycycline and tetracycline have different antibacterial potencies, but resistance genes and mechanisms are identical [16]. For these two pairs of antimicrobials, ECOFFs will identify identical non-wildtype susceptible populations. For the aminoglycosides neomycin and kanamycin, a variety of aminoglycoside-modifying enzymes can be involved [16]. In our experience, *E.coli* from animals are phenotypically mostly cross-resistant to these antimicrobials, but confirmation of absolute cross-resistance by typing of resistance genes is lacking.

Since 2008, the antimicrobial panel is decided by EFSA and included in EU legislation. In general, all antimicrobial classes of public health interest are represented in the panel. However, one of the disadvantages of using phenotypic methods is that the choice of specific antimicrobials is confined by the limited amount of wells in the Sensititre plates, to provide wide enough ranges for the tested substances. In the near future, phenotypic susceptibility testing for AMR surveillance in animals may be replaced by whole-genome sequencing, then any known resistance genes will be found.

### Statistical analysis

We considered several modelling methods for time-series data. An autoregressive integrated moving average (ARIMA) model was explored, which best fit data with high density of observations in short time-periods. The generalised additive model (GAM) with spline-functions applied by Boireau et al. is useful to correct for recurring trends such as seasonality [13]. In our study, monitoring data came from a standardised random sampling procedure with a relatively small yearly sample. This standardisation is one of the qualities of the programme, resulting in very little noise in the data. We therefore decided to use generalised linear models that allow for different distributions and chose not to use splines. Although splines are useful to form hypotheses about when and how many trend changes occurred [13], splines are not helpful in quantification of trends.

A Poisson distribution was preferred over binomial distribution, giving priority to trend assessment in emerging and rare resistances. Poisson has a high accuracy for low counts. In general, the Poisson distribution fitted the data better than the binomial distribution; AICs were lower. In the Poisson distribution the mean is equal to the variance. As can be seen from over or underdispersion, this assumption is not met for all antimicrobials. In these cases, data can be remodelled with other distributions, such as binomial (for high counts) or negative binomial distributions (for very low counts). The use of alternative distributions for over or underdispersion improved model fit for this data. However, the estimates were mostly similar, thus conclusions based on Poisson regression seem robust.

Poisson regression seems well suited for quantifying resistance time-trends over the past 20 years and to show trend changes as a result of interventions. However, when the aim is to compare recent monitoring data (a new year) with the previous years, this method may not be the most informative. Adding a new year of data to a time-series of multiple years will not affect estimates for time-trends of x1 and x2. Aberrations in new data can be detected by applying ‘year’ as a factor instead of a numerical in this Poisson regression model, showing separate estimates of each year relative to one reference year (data not shown).

In this study, we have investigated the best modelling approach to quantify trends over time and detect the effects of interventions within the current Dutch sampling frame. In the Technical specifications 2012 [22], EFSA has given recommendations (based on simulations) on how sampling strategy affects the power of detecting increases or decreases over time. Additional to the work in this study, it should be further investigated how different sample sizes or sampling intervals (every other year instead of a yearly sample) affect the ability of the monitoring programme to detect emerging resistances and trend changes.

### Relating AMR trends to AMU

AMR trends were independently quantified and effects of AMU regulations were reflected by the choice of the reference year in the model (2009). The EU monitoring programme in commensal *E. coli* from livestock aims to monitor the effects of AMU-interventions. Relating AMR trends to AMU at a national level is challenging in the first place, because not all member states have detailed data of veterinary AMU. Although there is an ecological correlation between resistant proportions of *E. coli* from animals at slaughter and AMU in livestock, as shown earlier by Dorado Garcia et al. [10], this correlation does not refer to individual animals. Only with extensive sampling at farm level, AMR trends from isolates in faecal samples from livestock can be directly correlated with AMU.

### Conclusion

This analysis of the standardised commensal *E. coli* dataset from the Dutch NRL for monitoring AMR in livestock, shows that monitoring in commensal *E. coli* is a useful tool to detect trends in phenotypic resistance in livestock relevant to public health (as defined by EFSA and EU legislation). We showed effective methods to quantify resistance trends in different antimicrobials and detect trend changes. The results of this study concern Dutch data, but this modelling approach is applicable to similar data acquired in other EU countries. The method can be applied to a dataset of any size, although the method will perform better when there is more data available.

## Supplementary Data

768000 Supplement S1
