# Supplementary material for: Monitoring antimicrobial resistance trends in commensal Escherichia coli from livestock, the Netherlands, 1998 to 2016
Source: Euro Surveill. 2019 Jun 20;24(25):1800438. doi: 10.2807/1560-7917.ES.2019.24.25.1800438 (PMC6593905; doi:10.2807/1560-7917.ES.2019.24.25.1800438)
Supplement: Supplement S1 [file 1800438_HESP_SupplementS1.pdf]

Supplement S1

"This supplementary material is hosted by *Eurosurveillance* as supporting information alongside the article 'Monitoring antimicrobial resistance trends in sentinel *Escherichia coli* from livestock, The Netherlands, 1998 to 2016' on behalf of the authors who remain responsible for the accuracy and appropriateness of the content. The same standards for ethics, copyright, attributions and permissions as for the article apply. *Eurosurveillance* is not responsible for the maintenance of any links or email addresses provided therein."

Contents

Table S1. MIC ranges used for susceptibility testing in Dutch monitoring program 1998 – 2016

Table S2. Data broilers: Counts of resistant *E. coli* isolates from Dutch monitoring 1998 - 2016

Table S3. Data slaughter pigs: Counts of resistant *E. coli* isolates from Dutch monitoring 1998 - 2016

Table S4. Data veal calves: Counts of resistant *E. coli* isolates from Dutch monitoring 2005 – 2016

Table S5. AMR-trends 1998-2016 for gentamicin, ciprofloxacin and nalidixic acid in indicator *E. coli* from slaughter pigs, modelled with negative binomial distribution

Table S1. MIC ranges used for susceptibility testing in Dutch monitoring program 1998 - 2016

Period of susceptibility testing	MIC ranges in mg/L for antimicrobials in panel															
	AMOX[a]	AMP	FOT	TAZ	GEN	DOX	TET	SMX	TMP	CHL	CIP	NAL	COL	FFN	KAN	NEO
1998 - 1999	0.06 - 64		0.015 - 16	0.03 - 32	0.03 - 32	0.03 - 32			0.03 - 32	0.06 - 64	0.004 - 32			0.06 - 64		
2001	0.5 - 32		0.12 - 8		0.25 - 32	0.25 - 32			0.5 - 64	2 - 128	0.03 - 8			2 - 128		1 - 128
2002	0.5 - 32		0.12 - 8		0.25 - 32	0.25 - 32			0.5 - 64	4 - 128	0.06 - 8			2 - 128		1 - 128
2003	0.5 - 32		0.12 - 8		0.25 - 32		0.5 - 64		0.5 - 64	4 - 128	0.06 - 8			2 - 128		1 - 128
2004 - 2005	0.5 - 64		0.12 - 16		0.25 - 32		0.5 - 64	8 - 1024	0.5 - 64	4 - 128	0.06 - 8	2 - 128		2 - 128		1 - 128
2005 - 2006	0.5 - 64		0.12 - 16	0.12 - 16	0.25 - 32		0.5 - 64	8 - 1024	0.5 - 64	4 - 128	0.06 - 8	2 - 128		2 - 128		1 - 128
first half 2007		0.5 - 64	0.12 - 16	0.12 - 16	0.25 - 32		0.5 - 64	8 - 1024	0.5 - 64	2 - 128	0.06 - 16	2 - 128		1 - 228		1 - 128
mid 2007 - 2010		0.5 - 32	0.06 - 4	0.25 - 16	0.25 - 32		1 - 64	8 - 1024	0.5 - 32	2 - 64	0.008 - 8	4 - 64	8 - 16	2 - 64	4 - 128	
2010 - 2013		0.5 - 32	0.06 - 4	0.25 - 16	0.25 - 32		1 - 64	8 - 1024	0.5 - 32	2 - 64	0.008 - 8	4 - 64	2 - 4	2 - 64	4 - 128	
2014 - 2016		1 - 64	0.25 - 4	0. 5 - 8	0.5 - 32		2 - 64	8 - 1024	0.25 - 32	8 - 128	0.015 - 8	4 - 128	1 - 16			

[a]AMOX, amoxicillin; AMP, ampicillin; FOT, cefotaxime; TAZ, ceftazidime; GEN, gentamicin; DOX, doxycycline; TET, tetracycline; SMX, sulfamethoxazole; TMP, trimethoprim; CHL, chloramphenicol; CIP, ciprofloxacin; NAL, nalidixic acid; COL, colistin; FFN, florfenicol; KAN, kanamycin; NEO, neomycin.

Table S2. Data broilers: Counts of resistant *E. coli* isolates from Dutch monitoring 1998 - 2016

**Year**	**1998**	**1999**	**2001**	**2002**	**2003**	**2004**	**2005**	**2006**	**2007**	**2008**	**2009**	**2010**	**2011**	**2012**	**2013**	**2014**	**2015**	**2016**
**Total isolates (N)**	**303**	**318**	**318**	**164**	**165**	**300**	**304**	**147**	**34**	**440**	**291**	**284**	**283**	**292**	**301**	**377**	**400**	**300**
	**Resistant isolates (n)**																	
Amoxicillin/ampicillin	118	113	164	85	82	193	193	96	19	288	213	217	187	204	170	234	213	141
Cefotaxime	8	2	5	12	5	29	43	24	7	66	52	52	23	17	8	11	10	3
Ceftazidime	5	3						23	7	64	51	50	23	18	8	12	10	3
Gentamicin	17	11	11	5	5	16	12	12	1	64	25	27	20	25	22	24	16	13
Doxycycline/tetracycline	168	204	187	97	99	200	185	79	18	256	180	170	146	148	124	160	143	91
Sulfamethoxazole						218	218	105	20	314	209	202	179	183	154	198	188	122
Trimethoprim	109	140	165	96	76	188	192	91	14	264	181	172	159	150	123	168	166	110
Chloramphenicol	15	20	34	27	26	69	55	28	5	111	69	74	58	48	44	51	44	21
Ciprofloxacin	117	114	131	74	59	136	155	72	17	268	167	181	156	147	164	175	176	123
Nalidixic acid						139	158	72	17	272	167	180	158	146	163	168	168	118
Florfenicol	2	1	0	3	0	6	3	1	1	21	12	9	11	4	4			
Neomycin/kanamycin			29	19	23	40	34	23	1	74	35	41	33	24	18			
Colistin												3	2	3	3	0	0	0

Table S3. Data slaughter pigs: Counts of resistant *E. coli* isolates from Dutch monitoring 1998 - 2016

**Year**	**1998**	**1999**	**2001**	**2002**	**2003**	**2004**	**2005**	**2006**	**2007**	**2008**	**2009**	**2010**	**2011**	**2012**	**2013**	**2014**	**2015**	**2016**
**Total isolates (N)**	**302**	**318**	**320**	**149**	**155**	**296**	**299**	**79**	**169**	**295**	**296**	**282**	**287**	**284**	**289**	**392**	**298**	**299**
	**Resistant isolates (n)**																	
Amoxicillin/ampicillin	50	44	60	38	43	77	91	27	60	105	133	94	102	71	68	94	86	69
Cefotaxime	2	2	1	2	1	2	2	0	1	3	11	2	5	0	5	2	1	1
Ceftazidime	2	1						0	1	4	11	2	7	0	4	4	1	1
Gentamicin	1	5	0	4	3	3	3	2	9	7	9	4	6	6	2	14	2	0
Doxycycline/tetracycline	145	167	151	86	107	189	185	55	124	201	200	179	192	160	152	193	135	128
Sulfamethoxazole						156	153	42	93	170	183	154	157	129	125	162	120	103
Trimethoprim	99	126	108	65	69	128	124	37	87	146	159	138	136	106	110	121	107	95
Chloramphenicol	24	39	16	14	12	36	26	8	21	31	34	33	35	33	30	47	28	38
Ciprofloxacin	1	5	4	1	0	10	0	1	3	5	21	1	5	3	0	0	2	2
Nalidixic acid						5	0	1	2	6	21	1	3	3	0	1	2	2
Florfenicol	0	0	0	0	1	4	0	0	1	3	2	4	2	3	5			
Neomycin/kanamycin			9	2	6	6	13	2	8	5	11	9	4	1	2			
Colistin												0	2	1	0	0	0	0

Table S4. Data veal calves: Counts of resistant *E. coli* isolates from Dutch monitoring 2005 - 2016

**Year**	**2005**	**2006**	**2007**	**2008**	**2009**	**2010**	**2011**	**2012**	**2013**	**2014**	**2015**	**2016**
**Total isolates (N)**	165	152	175	163	171	172	166	285	317	292	293	300
**Resistant isolates (n)**												
Ampicillin	80	73	82	65	71	73	81	73	79	65	55	70
Cefotaxime	5	3	7	4	3	3	5	2	1	3	0	1
Ceftazidime	1	4	4	3	4	3	4	0	0	2	0	1
Gentamicin	21	17	11	20	11	18	19	7	5	11	2	10
Tetracycline	136	110	124	109	101	125	122	137	153	130	119	124
Sulfamethoxazole	90	83	92	72	77	91	93	93	101	82	69	65
Trimethoprim	73	67	75	69	64	75	75	70	75	65	38	60
Chloramphenicol	55	42	51	31	38	50	44	41	43	39	34	45
Ciprofloxacin	42	28	32	33	31	33	39	16	27	19	10	11
Nalidixic acid	43	28	31	33	32	30	37	15	28	17	9	5
Florfenicol	30	15	18	20	12	38	28	21	17			
Neomycin/kanamycin	45	38	25	39	28	31	46	16	24			
Colistin						9	9	2	3	0	0	0

Table S5. AMR-trends 1998-2016 for gentamicin, ciprofloxacin and nalidixic acid in indicator *E. coli* from slaughter pigs, modelled with negative binomial distribution

**Antimicrobial**	**Variable**	**Estimate**	**P (>|z|)**[d]	**IRR**[e]	**AIC**[f]	**Scaled deviance**[g] (0.5 <> 2)
Gentamicin	Intercept[a]	-3.40	0.00	0.03	93.63	1.40
	x1[b]	0.14	0.01	1.15		
	x2[c]	-0.19	0.03	0.82		
Ciprofloxacin	Intercept	-3.61	0.00	0.03	86.48	1.34
	x1	0.12	0.11	1.13		
	x2	-0.33	0.02	0.72		
Nalidixic acid*	Intercept	-3.56	0.00	0.03	63.98	1.39
	x1	0.25	0.16	1.29		
	x2	-0.33	0.01	0.72		

*For nalidixic acid data were not collected during whole length of testing period, see supplementary data

[a] Intercept, estimated value for reference year 2009

[b] x1, time in years before reference year 2009

[c] x2, time in years since reference year 2009

[d] P (>|z|), p-values <0.05 indicate significant trends for variables x1 and x2

[e] Incidence Rate Ratio (IRR) with 95% confidence interval, for the intercept this number indicates the estimated resistant proportion for reference year 2009. For variables x1 and x2 this is the mean increase or decrease per year

[f] AIC, Akaike's Information Criterion, a measure for the fit of the model, a lower value indicates a better fit

[g] Scaled deviance, deviance divided by degrees of freedom, measure for dispersion, ideally this is 1. Scaled deviance of >2 indicates overdispersion of data, scaled deviance of <0.5 indicates underdispersion of data.
